# Investigation of synovial fluid lubricants and inflammatory cytokines in the horse: a comparison of recombinant equine interleukin 1 beta-induced synovitis and joint lavage models

**DOI:** 10.1186/s12917-021-02873-2

**Published:** 2021-05-12

**Authors:** Amanda Watkins, Diana Fasanello, Darko Stefanovski, Sydney Schurer, Katherine Caracappa, Albert D’Agostino, Emily Costello, Heather Freer, Alicia Rollins, Claire Read, Jin Su, Marshall Colville, Matthew Paszek, Bettina Wagner, Heidi Reesink

**Affiliations:** 1 Department of Clinical Sciences, College of Veterinary Medicine, Cornell University, Ithaca, NY USA; 2 Department of Biostatistics, School of Veterinary Medicine, University of Pennsylvania, Philadelphia, PA USA; 3 Robert Frederick Smith School of Chemical and Biomolecular Engineering, Cornell University, Ithaca, NY USA; 4 Department of Population Medicine and Diagnostic Sciences, College of Veterinary Medicine, Cornell University, Ithaca, NY USA

**Keywords:** Repeated arthrocentesis, Lubricin, Hyaluronic acid, Chemokine, Rheology, Lubrication, Osteoarthritis

## Abstract

**Background:**

Lameness is a debilitating condition in equine athletes that leads to more performance limitation and loss of use than any other medical condition. There are a limited number of non-terminal experimental models that can be used to study early inflammatory and synovial fluid biophysical changes that occur in the equine joint. Here, we compare the well-established carpal IL-1β-induced synovitis model to a tarsal intra-articular lavage model, focusing on serial changes in synovial fluid inflammatory cytokines/chemokines and the synovial fluid lubricating molecules lubricin/proteoglycan 4 and hyaluronic acid. The objectives of this study were to evaluate clinical signs; synovial membrane and synovial fluid inflammation; and synovial fluid lubricants and biophysical properties in response to carpal IL-1β synovitis and tarsal intra-articular lavage.

**Results:**

Hyaluronic acid (HA) concentrations, especially high molecular weight HA, and synovial fluid viscosity decreased after both synovitis and lavage interventions. Synovial fluid lubricin concentrations increased 17–20-fold for both synovitis and lavage models, with similar changes in both affected and contralateral joints, suggesting that repeated arthrocentesis alone resulted in elevated synovial fluid lubricin concentrations. Synovitis resulted in a more severe inflammatory response based on clinical signs (temperature, heart rate, respiratory rate, lameness and joint effusion) and clinicopathological and biochemical parameters (white blood cell count, total protein, prostaglandin E_2_, sulfated glycosaminoglycans, tumor necrosis factor-α and CC chemokine ligands − 2, − 3, − 5 and − 11) as compared to lavage.

**Conclusions:**

Synovial fluid lubricin increased in response to IL-1β synovitis and joint lavage but also as a result of repeated arthrocentesis. Frequent repeated arthrocentesis is associated with inflammatory changes, including increased sulfated glycosaminoglycan concentrations and decreased hyaluronic acid concentrations. Synovitis results in more significant inflammatory changes than joint lavage. Our data suggests that synovial fluid lubricin, TNF-α, CCL2, CCL3, CCL5, CCL11 and sGAG may be useful biomarkers for synovitis and post-lavage joint inflammation. Caution should be exercised when performing repeated arthrocentesis clinically or in experimental studies due to the inflammatory response and loss of HA and synovial fluid viscosity.

**Supplementary Information:**

The online version contains supplementary material available at 10.1186/s12917-021-02873-2.

## Background

Lameness due to joint disease is a major cause of decreased performance and loss of use in horses [1, 2]. Treatment can range from systemic medications and nutraceuticals to intra-articular treatment or arthroscopic evaluation, depending upon the nature and severity of the injury. Treatment with systemic non-steroidal anti-inflammatories and intra-articular therapies, including steroids and lubricants, are mainstays of treatment for synovitis and early osteoarthritis (OA) as these drugs interrupt the inflammatory cascade and provide viscosupplementation [1, 3–5]. Biologic therapies, such as platelet rich plasma (PRP) or PRP lysate, are also increasingly being used [4, 6, 7]. Early treatment of synovitis is critical to slow the progression of OA by combating inflammation-induced changes in synovial fluid composition and viscosity. Arthroscopic lavage has been used to treat OA and is commonly used to diagnose and treat intra-articular fracture, cartilage damage or idiopathic synovitis [8–11]. There are few non-terminal equine experimental joint injury models that can be used to evaluate changes in synovial fluid composition and response to therapeutics. Intra-articular lavage and interleukin 1 beta (IL-1β)-induced synovitis are two transient experimental models used to mimic clinical synovitis and study early joint disease in a controlled manner without the requirement for euthanasia.

Arthroscopic lavage decreases inflammatory cytokines, including IL-1β and tumor necrosis factor alpha (TNF-α), thereby mitigating synovitis and cartilage degradation in rabbits with experimentally-induced stifle OA [8]. A canine cartilage explant study investigated the effect of irrigation fluid osmolality on chondrocyte death and proteolytic gene expression and found no effect on cell death and a decrease in protease gene expression following exposure to all fluid groups [12]. In an ex vivo bovine stifle joint study, articular cartilage surface friction increased following arthroscopic lavage, and lubricin cartilage immunostaining was reduced as compared to non-lavage controls [13]. In equids, arthroscopic lavage is primarily used for the diagnosis and treatment of joint pathology, including septic arthritis, intra-articular fragment removal and treatment of focal cartilage defects and subchondral bone disease [14]. An in vivo equine study comparing gas and liquid arthroscopy in the tarsocrural joint revealed that prostaglandin E_2_ (PGE_2_) was increased following both gas distension and intra-articular lavage [15]. This data suggests that, while arthroscopic lavage can be therapeutic by removing harmful cytokines in diseased joints, the lavage itself can incite inflammation in healthy joints.

In the healthy joint, hyaluronic acid (HA) and lubricin/proteoglycan 4 work in concert to decrease friction and surface wear between the articular cartilage surfaces and between articular cartilage and soft tissues of the joint such as menisci, enabling pain free movement [16–18]. The glycosylation of synovial fluid lubricin has been shown to differ between osteoarthritic and healthy equine joints, which may lead to decreased lubricating ability in diseased joints [19]. Following arthroscopy of equine middle carpal joints, synovial fluid viscosities and HA concentrations decrease for up to 75 days [20]. Bovine cartilage explants, lubricated with lubricin-deficient synovial fluid from humans with camptodactyly-arthropathy-coxa vara-pericarditis syndrome (CACP), an arthritis-like autosomal recessive disorder, reveal increased friction coefficients and increased chondrocyte apoptosis as compared to normal synovial fluid or CACP synovial fluid to which lubricin has been added [21]. These changes lead to cartilage degradation and perpetuation of inflammation, highlighting the importance of lubricin’s cartilage lubricating function [12]. To avoid these detrimental cartilage effects, synovial fluid (SF) substitutes have been developed for use in humans in the postoperative period. The use of an intra-articular HA product decreased reported postoperative pain and NSAID usage in a single blind randomized controlled pilot study in partial meniscectomy patients. In addition, this intra-articular HA-based synovial fluid substitute decreased patient-reported pain measures for up to 1 year in a prospective, randomized, double-blinded study following knee arthroscopy and cartilage debridement to treat chronic knee pain [22, 23]. To the authors’ knowledge, the effects of joint lavage on SF lubricants have not been previously studied in horses.

Synovitis is characterized clinically by joint swelling and effusion [24]. Synovitis models are well established in horses, with lipopolysaccharide (LPS) [25–29] and IL-1β [30–33] being the most frequently used, and IL-1β-induced synovitis considered a more appropriate model for early OA due to the known contribution of IL-1β to OA pathogenesis [32]. In human patients presenting to an emergency room with acute knee joint synovitis, synovial fluid aspirated from synovitis knees had inferior lubrication properties as compared to healthy synovial fluid [34]. In a comparison between inflammatory and non-inflammatory OA SF as defined by a white blood cell count higher than 2000 cells/mm^3^_,_ inflammatory SF caused increased tissue strain on cartilage explants, resulting in increased chondrocyte death and apoptosis [35]. Cartilage breakdown products elicit the release of proinflammatory and catabolic mediators from synovial membrane and cartilage, including IL-1β, TNF-α, matrix metalloproteases (MMPs), and prostaglandin E_2_ (PGE_2_) and many others which propagate the inflammation and exacerbate joint deterioration [24]. IL-1β and TNF-α have been shown to increase acutely following traumatic injury of the fetlock in Standardbred racehorses and remain elevated for 3 years, while TNF-α was predictive of radiographic progression of fetlock OA [36]. An equine model of amphotericin B-induced synovitis showed a delayed but sustained elevation of IL-1β and TNF-α over the 9-week study period [37].

Chemokines are a large group of molecules that are divided into families based on the relative positions of their cysteine residues [38]. All chemokine receptor ligands recruit immune cells to areas of inflammation, but the CC chemokine receptor ligand family with two adjacent cysteine residues (CCLs) such as CCL2, CCL3, CCL5, and CCL11 mainly act on monocytes and lymphocytes [39–42]. Recently developed assays to measure CCL concentrations suggest that these molecules have potential as pro-inflammatory biomarkers based on their presence on stimulated immune cells and their activation of macrophages into pathways that stimulate anti-microbial and tumor activity and promote immunoregulation and tissue healing [43, 44]. Chemokines are increased in diverse inflammatory diseases [40, 42, 45] and recruit inflammatory cells to bone and connective tissue in rheumatoid arthritis [45]. In addition to certain subsets of chemokines being reduced in OA and rheumatoid arthritis compared to healthy synovial fluid while other subsets are elevated in these groups, certain CC chemokines in synovial fluid influence the migration of human mesenchymal cell progenitors which could affect cartilage healing following joint injury [46]. Chemokines have been minimally investigated in equine joint disease to date.

Given the importance of synovial fluid inflammatory biomolecules and lubricants in joint health and OA progression, our objective was to compare clinical, biochemical and synovial fluid biophysical parameters in the equine IL-1β-induced carpal synovitis model and intra-articular tarsocrural lavage model. We hypothesized that lubricin and HA concentrations would decrease in both models and that both models would result in transient, self-limiting joint inflammation associated with measurable increases in pro-inflammatory cytokines/chemokines.

## Results

**Fig. 1:**
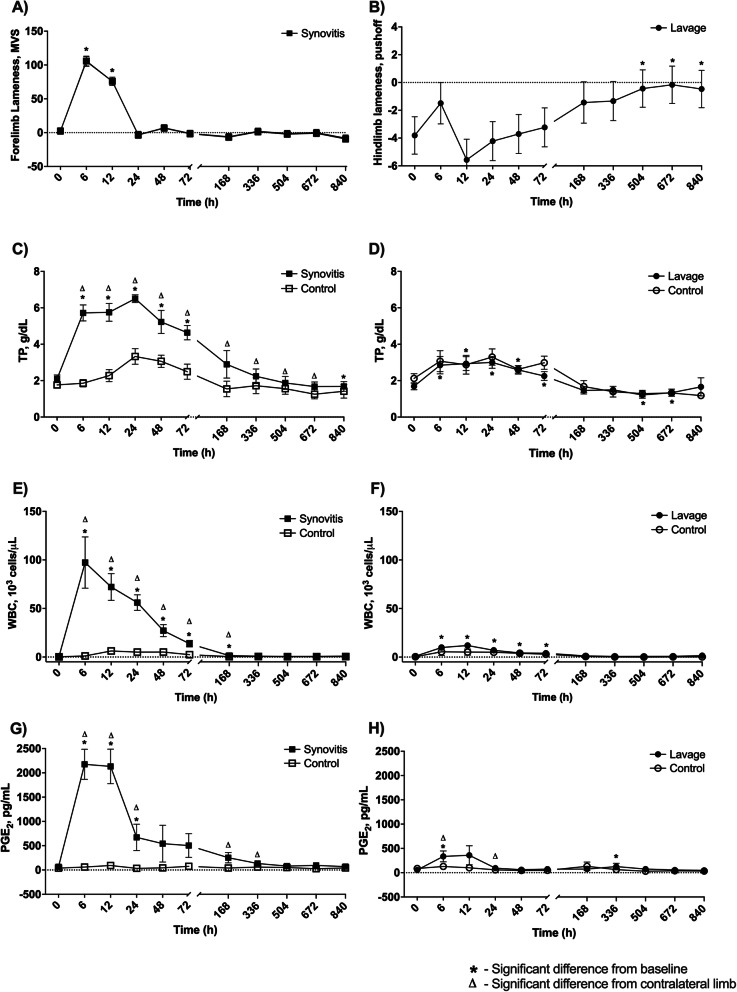
Lameness scores and total protein (TP), white blood cell (WBC), and prostaglandin E_2_ (PGE_2_) concentrations in the synovial fluid of middle carpal joints (MCJ) and tarsocrural joints (TCJ) following IL-1β-induced synovitis or intra-articular lavage. Forelimb lameness, as measured by mean vector sum using an inertial motion sensor system, increased markedly from baseline for 12 h post-induction of synovitis on the synovitis limb (**a**). Hind limb lameness as measured by pushoff factor showed that horses started the study with a trend toward mild lameness of the contralateral hindlimb (negative values) in the first week but became sound (− 2 to + 2) in the last 4 weeks of the study (**b**). TP increased from baseline for 72 h following synovitis induction and was greater in synovitis MCJ as compared to contralateral MCJ for 4 weeks post-synovitis induction. TP decreased from baseline at 5 weeks post-induction (**c**). TP increased from baseline for 72 h following lavage with no differences from contralateral TCJ. TP decreased from baseline at 3 and 4 weeks post-lavage (**d**). WBC counts increased from baseline and were greater in synovitis MCJ as compared to contralateral MCJ for 1 week post-synovitis induction (**e**). WBC counts increased from baseline for 72 h following lavage with no differences from contralateral TCJ (**f**). PGE_2_ increased from baseline for 24 h following synovitis and was greater in synovitis MCJ as compared to contralateral MCJ for the majority of 2 weeks post-synovitis induction (**g**). Following lavage, PGE_2_ increased from baseline at 6 h and was greater in lavage TCJ as compared to contralateral TCJ at 6 and 24 h post-lavage (**h**). An asterisk indicates a difference from baseline, and a triangle indicates a difference from the contralateral limb at the same time point. Graphed values are marginal means +/− SEM

Six horses (3 mares and 3 geldings) from 8 to 20 years of age (mean: 14.5 years, median: 15 years) and weights ranging from 511 to 636 kg (mean: 570 kg, median: 568.2 kg) were included in this study. IL-1β-induced synovitis resulted in moderate, transient increases in heart rate (HR) and rectal temperature (T) (synovitis peak HR: 52 ± 2 beats per minute at 12 h, *p* = 0.0001; synovitis peak T: 101.1 ± 0.2 °F at 12 h, *p* = 0.0001), while lavage resulted in a slight increase in rectal temperature at 12 h (lavage peak T: 99.6 ± 0.1 °F, *p* = 0.001) and no increases in HR (Supplemental Data 1A-D). Respiratory rate (RR) was unchanged post-synovitis and decreased at 6–12 h post-lavage (lavage trough RR: 12 ± 2.4 breaths per minute at 6 h, *p* = 0.015) (Supplemental Data 1E-F). Induction of synovitis in the middle carpal joint resulted in an increase in joint circumference (JC) from baseline from 12 to 48 h and at 1, 2, 3, and 4 weeks (synovitis peak JC: 1.1 times baseline at 48 h, *p* = 0.0001), and the synovitis MCJ was increased in circumference from the contralateral MCJ from 12 to 48 h and at 5 weeks at 48 h (*p* = 0.0001). Intra-articular lavage of the tarsocrural joint did not change joint circumference (Supplemental Data 1G-H). Synovitis resulted in a severe but temporary lameness on the synovitis limb that returned to baseline by 24 h post-induction (mean vector sum baseline: 2 ± 4, synovitis mean vector sum peak: 105 ± 7, *p* = 0.001) (Fig. 1a). Horses began the study with a slight predisposition for pushoff lameness on the contralateral hindlimb prior to lavage (negative value) and progressed to soundness (from − 2 to + 2) at 168 h post lavage (pushoff value baseline: − 3.8 ± 1, lavage pushoff peak: − 0.16 ± 1, *p* = 0.006) (Fig. 1b).

Synovial fluid clinicopathological and biochemical parameters were used to both validate the IL-1β synovitis model and to compare inflammatory changes between the IL-1β synovitis and lavage models (Fig. 1c-h and Supplemental Data 2). Total protein (TP) and white blood cell concentration (WBC) increased following induction of both models for 72 h, with a greater magnitude of increase in the synovitis model (peak TP synovitis: 6.5 ± 0.2 g/dL, *p* = 0.0001; peak TP lavage: 3 ± 0.3 g/dL, *p* = 0.0001); peak WBC synovitis: 97,200 ± 26,400 cells/μL, p = 0.0001; peak WBC lavage: 11,800 ± 3000 cells/μL, *p* = 0.001) (Fig. 1c-f). Synovitis induction resulted in significant increases in PGE_2_ from baseline at 6, 12, and 24 h with differences from the sham-injected control limb at 6, 12, 24, 168, and 336 h (synovitis PGE_2_ baseline: 67 ± 27 pg/mL; synovitis PGE_2_ peak: 2176 ± 310 pg/mL, *p* = 0.0001). Lavage only resulted in slight increases in PGE_2_ concentration from baseline at 6 and 336 h post-lavage with differences between lavage and control limbs only detected at 6 and 24 h post-lavage (lavage PGE_2_ baseline: 55 ± 48 pg/mL; lavage PGE_2_ peak: 335 ± 112 pg/mL, *p* = 0.046). The synovitis model PGE_2_ concentrations were significantly higher than the lavage model at 6, 12, 24, and 72 h post-induction (*p* = 0.0001).

**Fig. 2:**
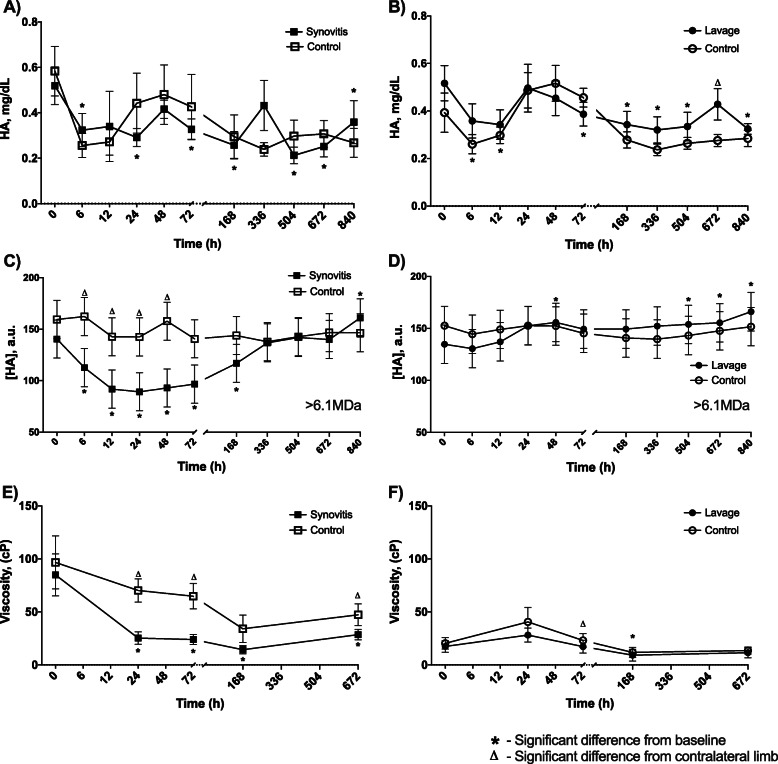
Hyaluronic acid (HA) concentration, relative concentration of HA with a molecular weight of > 6.1 MDa (HMW HA), and viscosity of the synovial fluid of middle carpal joints (MCJ) and tarsocrural joints (TCJ) following IL-1β-induced synovitis or intra-articular lavage. HA decreased from baseline at 6, 24, and 72 h and at 1, 3, 4, and 5 weeks following synovitis induction in both synovitis MCJ and contralateral MCJ (**a**). HA decreased from baseline from 6 to 12 h, 72 h to 3 weeks, and at 5 weeks post-lavage in both lavage TCJ and contralateral TCJ (**b**). HMW HA decreased from baseline in synovitis MCJ for 1 week post-synovitis induction and was less than control MCJ from 6 to 48 h. HMW HA increased above baseline at 5 weeks (**c**). HMW HA increased from baseline at 48 h and from 3 to 5 weeks post-lavage with no statistical differences from contralateral TCJ (**d**). Viscosity decreased from baseline for the duration of the study period (4 weeks) following synovitis induction and was less than the contralateral MCJ for the same period (except at 1 weeks) (**e**). Following lavage, viscosity was unchanged from baseline except for a decrease at 168 h and was less than the contralateral TCJ at 72 h (**f**). Baseline viscosity between the MCJ and TCJ were statistically different. An asterisk indicates a difference from baseline, and a triangle indicates a difference from the contralateral limb at the same time point. Graphed values are marginal means +/− SEM

The effects of each model on synovial fluid HA concentration and viscosity were evaluated (Fig. 2 and Supplemental Data 2). Following synovitis, HA concentration decreased from baseline at 6, 24, 72, 168, 504, and 840 h with differences between control and synovitis joints at 336 h only (synovitis HA baseline: 0.5 ± 0.1 mg/mL; synovitis HA trough: 0.2 ± 0.03 mg/mL, *p* = 0.0001). HA concentration also decreased following lavage at 6, 12, 72, 168, 336, 504 and 840 h with differences between control and lavage joints present only at 672 h (lavage HA baseline: 0.5 ± 0.1 mg/mL; lavage HA trough: 0.32 ± 0.05 mg/mL, *p* = 0.0001) (Fig. 2a-b). The proportion of high molecular weight HA (> 6 MDa) decreased from baseline from 6 to 168 h post-induction of synovitis and was decreased from the contralateral limb from 6 to 48 h (*p* = 0.0001). The lavage model resulted in an increased proportion of high molecular weight HA from baseline at 48, 504, 672, and 840 h (*p* = 0.001). There were no differences in the proportion of high MW HA between intervention and control limbs following lavage (Fig. 2c-d). Viscosity was decreased following induction of synovitis for the duration of the study period and was significantly decreased from the contralateral sham-injected limb at 24, 72, and 672 h (synovitis viscosity baseline: 85 ± 20 cP; synovitis viscosity trough: 14 ± 4 cP, *p* = 0.002). Viscosity remained fairly stable following lavage, with decreased viscosity noted in the lavage limb at 168 h only, and a decreased viscosity in the lavage joint as compared to the contralateral joint at 72 h (lavage viscosity baseline: 17 ± 5 cP; lavage viscosity trough 9 ± 5.5 cP, *p* = 0.004). The middle carpal joint (MCJ) had significantly higher viscosity than the tarsocrural joint (TCJ) at baseline (*p* = 0.007) (Fig. 2e-f) [47].

**Fig. 3:**
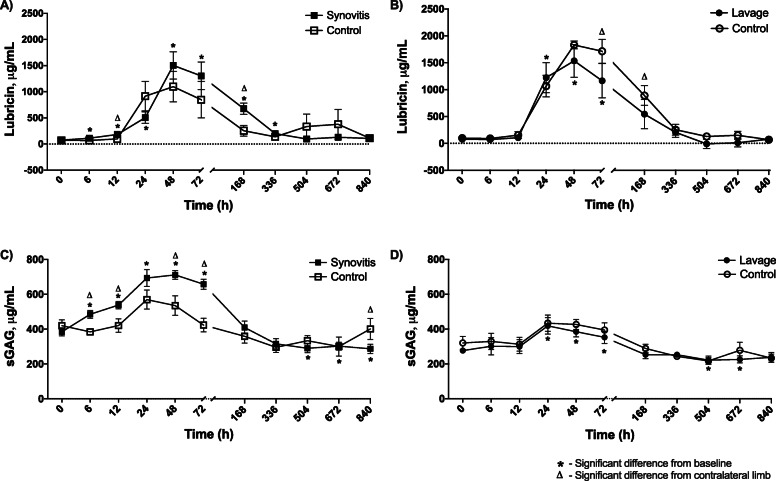
Lubricin and sulfated glycosaminoglycan (sGAG) concentrations in the synovial fluid of middle carpal joints (MCJ) and tarsocrural joints (TCJ) following IL-1β-induced synovitis or intra-articular lavage. Lubricin increased from baseline for 2 weeks following synovitis induction in both synovitis and contralateral MCJ (**a**). Lubricin increased from baseline for 72 h following lavage with the contralateral TCJ concentration being greater than synovitis TCJ at 72 and 168 h post-lavage (**b**). sGAG concentration increased from baseline for 72 h and was greater in synovitis MCJ as compared to contralateral MCJ during that time. sGAG concentration decreased from baseline at 3, 4 and 5 weeks post-induction of synovitis (**c**). sGAG concentration increased from baseline from 24 to 72 h following lavage and decreased from baseline 3 and 4 weeks with no differences from contralateral TCJ (**d**). An asterisk indicates a difference from baseline, and a triangle indicates a difference from the contralateral limb at the same time point. Graphed values are marginal means +/− SEM

The effects of each model on synovial fluid lubricin and sulfated glycosaminoglycan (sGAG) concentrations were evaluated using a sandwich ELISA and dimethylmethylene blue (DMMB) assay, respectively (Fig. 3a-d and Supplemental Data 2). Lubricin increased markedly in all sampled joints regardless of treatment, with infrequent differences between the intervention and control joints (synovitis lubricin baseline: 74 ± 17 μg/mL; synovitis lubricin peak: 1504 ± 260 μg/mL, *p* = 0.0001; lavage lubricin baseline: 77 ± 27 μg/mL; lavage lubricin peak: 1535 ± 307 μg/mL, *p* = 0.0001) suggesting that repeated arthrocentesis may have been the primary determinant of this response (Fig. 3a-b). Lubricin concentrations peaked at 48 h post-induction for both models and decreased as the arthrocentesis intervals increased from 1 week on. Sulfated glycosaminoglycan concentration increased in response to both models, albeit to a greater extent in synovitis (Fig. 3c-d). Following induction of synovitis, the sGAG concentration remained elevated from 6 to 72 h post-induction, with significant differences from the sham-injected joint at 12, 24, 48, 72 and 840 h (synovitis sGAG baseline: 385 ± 23 μg/mL; synovitis sGAG peak: 711 ± 24 μg/mL, *p* = 0.0001). The lavage model resulted in an increase in sGAG concentration from 24 to 72 h post-lavage and a decrease in concentration from baseline at 504 and 672 h post-lavage with no differences between lavage and control joints (lavage sGAG baseline: 276 ± 9 μg/mL; lavage sGAG peak: 418 ± 48 μg/mL, *p* = 0.002). The MCJ had an increased concentration of sGAG as compared to the TCJ at baseline (*p* = 0.0001), and the sGAG concentration remained elevated in the MCJ compared to the TCJ for the entire study period.

**Fig. 4:**
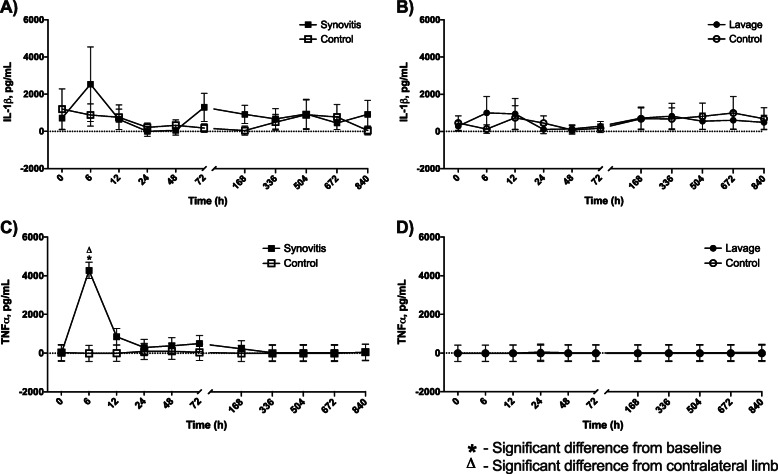
Interleukin-1β (IL-1β) and tumor necrosis factor α (TNF-α) concentrations in the synovial fluid of middle carpal joints (MCJ) and tarsocrural joints (TCJ) following IL-1β-induced synovitis or intra-articular lavage. IL-1β remained unchanged following synovitis induction (**a**) or intra-articular lavage (**b**). TNF-α was increased from baseline and was greater in synovitis MCJ as compared to contralateral MCJ at 6 h post-synovitis induction (**c**). TNF-α was unchanged from baseline following lavage with no differences from contralateral TCJ (**d**). An asterisk indicates a difference from baseline, and a triangle indicates a difference from the contralateral limb at the same time point. Graphed values are marginal means +/− SEM

**Fig. 5:**
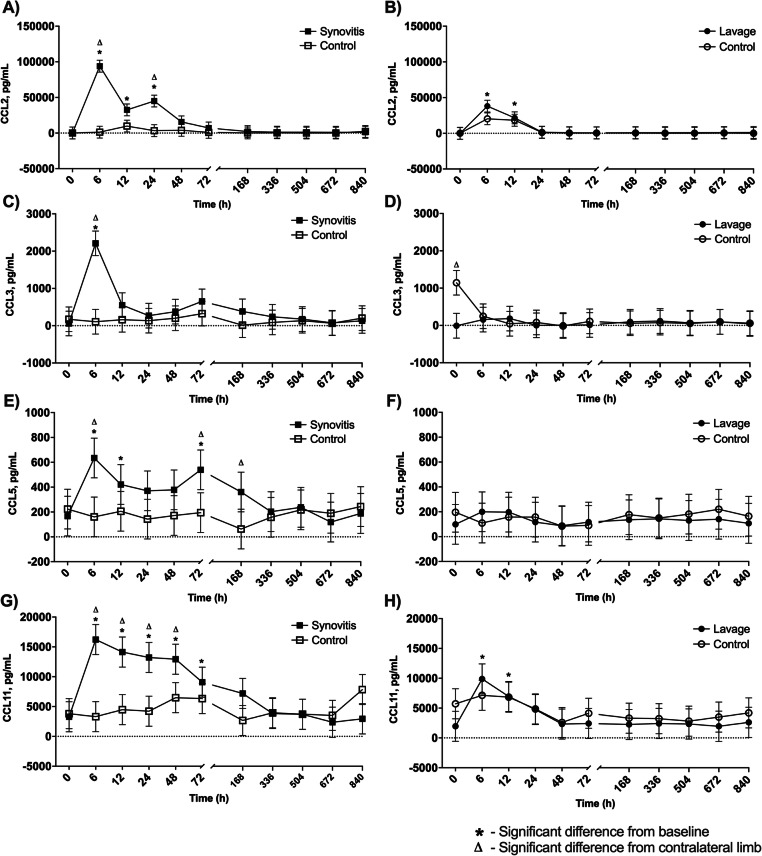
Chemokine ligand 2 (CCL2), chemokine ligand 3 (CCL3), chemokine ligand 5 (CCL5) and chemokine ligand 11 (CCL11) concentrations in the synovial fluid of middle carpal joints (MCJ) and tarsocrural joints (TCJ) following IL-1β-induced synovitis or intra-articular lavage. CCL2 was increased from baseline from 6 to 24 h following synovitis and was greater in synovitis MCJ as compared to contralateral MCJ at 6 and 24 h post-synovitis induction (**a**). Following lavage, CCL2 was increased from baseline from 6 to 12 h but did not differ from contralateral TCJ (**b**). CCL3 was increased from baseline and was greater in synovitis MCJ compared to contralateral MCJ at 6 h post-synovitis induction (**c**). CCL3 was less in the lavage TCJ as compared to the contralateral TCJ at baseline but otherwise had no response to lavage (**d**). Following synovitis, CCL5 was increased from baseline at 6, 12, and 72 h and was greater compared to the contralateral MCJ at 6, 72, and 168 h (**e**). CCL5 did not change in response to lavage (**f**). CCL11 was increased from baseline from 6 to 72 h following synovitis and was greater in synovitis MCJ than contralateral MCJ from 6 to 48 h (**g**). CCL11 was increased from baseline at 6 and 12 h post-lavage and did not differ from contralateral TCJ at any time. An asterisk indicates a difference from baseline, and a triangle indicates a difference from the contralateral limb at the same time point. Graphed values are marginal means +/− SEM

A fluorescent bead-based multiplex assay was performed to assess synovial fluid inflammatory cytokine and chemokine changes induced by each model (Figs. 4a-b and 5a-h and Supplemental Data 2). IL-1β concentration did not vary from baseline or between the control and affected joint following induction of either model, although a non-significant peak was present at 6 h post-induction of both synovitis and lavage (synovitis IL-1β baseline: 715 ± 620 pg/mL; synovitis IL-1β peak: 2536 ± 2004 pg/mL at 6 h; lavage IL-1β baseline: 286 ± 269 pg/mL; lavage IL-1β peak: 1001 ± 888 pg/mL) (Fig. 4a-b). TNF-α concentration increased from baseline at 6 h post-induction of synovitis and was greater than the contralateral sham-injected MCJ at 6 h post-induction (synovitis TNF-α baseline: − 6 ± 421 pg/mL; synovitis TNF-α peak: 4276 ± 421 pg/mL, *p* = 0.0001; lavage TNF-α baseline: − 10 ± 421 pg/mL; lavage TNF-α peak: 49 ± 421 pg/mL) (Fig. 4c). TNF-α was not altered by intra-articular lavage (Fig. 4d). An increase in CCL2 concentration was seen at 6, 12, and 24 h post-induction of synovitis with differences from the contralateral limb at 6 and 24 h. Following lavage, CCL2 was increased from baseline at 6 and 12 h (synovitis CCL2 baseline: 146 ± 8337 pg/mL; synovitis CCL2 peak: 93,869 ± 8335 pg/mL, *p* = 0.0001; lavage CCL2 baseline: − 123 ± 8337 pg/mL; lavage CCL2 peak: 37,993 ± 8335 pg/mL, *p* = 0.001) (Fig. 5a-b). An increase in CCL3 concentration from baseline was seen at 6 h post-induction of synovitis, with an increase in CCL3 in the synovitis as compared to control MCJ at 6 h (synovitis CCL3 baseline: 59 ± 330 pg/mL; synovitis CCL3 peak: 2209.5 ± 330 pg/mL (*p* = 0.0001)) (Fig. 5c). There were no differences from baseline or control limb in CCL3 concentration post-lavage; however, CCL3 concentrations were greater in control as compared to lavage TCJ at baseline (lavage CCL3 baseline: − 8 ± 330 pg/mL; lavage CCL3 peak: 183 ± 331 pg/mL) (Fig. 5c-d). Following synovitis, CCL5 concentrations were increased from baseline at 6, 12, and 72 h and were greater in the synovitis joint as compared to the contralateral MCJ at 6, 72, and 168 h (synovitis CCL5 baseline: 167 ± 160 pg/mL; synovitis CCL5 peak: 634 ± 159 pg/mL, *p* = 0.0001) (Fig. 5e). CCL5 concentrations were not altered by intra-articular lavage (lavage CCL5 baseline: 99 ± 160 pg/mL; lavage CCL5 peak: 200 ± 160 pg/mL) (Fig. 5f). CCL11 was increased from baseline at 6–72 h post-synovitis and at 6 and 12 h post-lavage (synovitis CCL11 baseline: 3288 ± 2521 pg/mL; synovitis CCL11 peak: 16,225 ± 2520 pg/mL, *p* = 0.0001; lavage CCL11 baseline: 1951 ± 2521 pg/mL; lavage CCL11 peak: 9883 ± 2520 pg/mL, *p* = 0.002) (Fig. 5g-h). CCL11 concentration was greater in the synovitis MCJ than the contralateral MCJ at 6–48 h (*p* = 0.0001), and there were no differences between limbs following lavage.

In order to assess inflammatory gene expression and expression of the genes encoding for hyaluronic acid synthases (*HAS1, HAS2, HAS3*), lubricin (proteoglycan 4, *PRG4)*, TNF-α stimulated gene 6 (*TSG6*) and IL-1β (*IL1β*), quantitative RT-PCR analysis of synovial fluid cell pellets was performed at 12 and 24 h post-induction of synovitis or lavage and of synovial membrane biopsies at 5 weeks post-induction of synovitis or lavage. Genes and primer sequences are available in Supplemental Data 3. *TSG6* expression was increased in synovial fluid cell pellets from the synovitis MCJ as compared to the lavage TCJ at 12 h and 24 h post-induction (*p* = 0.0001). At 24 h post-induction, *TSG6* expression was greater in the synovitis MCJ than the contralateral, sham-injected MCJ (*p* = 0.013). At 12 h post-induction of synovitis, both HA synthase 1 (*HAS1*) (*p* = 0.024) and HA synthase 3 (*HAS3*) (*p* = 0.01) expression were decreased in the cell pellets of the synovitis MCJ as compared to the lavage TCJ. There were no differences between groups in synovial fluid cell pellet *PRG4* expression. In the synovial membrane samples, there were no differences between groups in *HAS2*, *IL-1β* or *PRG4* expression. There were no histological differences in vascularity, inflammatory cellular infiltration, intimal hyperplasia, subintimal edema, and subintimal fibrosis between synovitis and lavage or between intervention and control limbs.

## Discussion

Both IL-1β-induced synovitis and intra-articular lavage resulted in transient joint inflammation with elevations in synovial fluid TP, WBC count, and PGE_2_; however, increases in TNF-α and inflammatory chemokines CCL2, CCL3, CCL5, and CCL11 were predominantly restricted to synovitis joints only. Synovial fluid sGAG and lubricin increased significantly in both intervention and control joints in both synovitis and lavage models, accompanied by a loss of HA, especially high MW HA, in both intervention and control joints. Likewise, synovial fluid viscosity decreased in both intervention and control joints, albeit to a greater extent in synovitis than lavage joints. This is the first report to demonstrate an increase in synovial fluid lubricin following either IL-1β-induced synovitis or intra-articular lavage, contrary to our hypotheses. The inflammatory changes observed suggest that both models induce transient inflammation, more pronounced in the synovitis model, and that either a systemic effect, or more likely repeated arthrocentesis may contribute to inflammation and changes in lubricating molecules in both the intervention and control joints.

Increased synovial fluid lubricin has been observed in several experimental and naturally occurring equine models of joint disease, including carpal osteochondral fragmentation, tarsal impact injury, full-thickness cartilage defect injury and spontaneous osteochondral fragmentation and osteoarthritis [47–49]. Interestingly, in prior studies investigating the equine carpal osteochondral fragment high-speed treadmill exercise model, synovial fluid lubricin concentrations were increased in both the fracture joint and, to a lesser extent, in the sham-operated control joint, possibly due to compensatory overloading of the control limb [20, 48]. Results from the current study may be able to help account for the findings in the sham-operated control joint, as these carpi undergo lavage during the arthroscopic sham-operation and are subjected to repeated arthrocentesis at weekly or more frequent intervals. LPS-induced equine synovitis models have shown decreased HA over a 48 h period, and a quantitative intercompartmental synovial joint model predicted that HA would take longer to reach a steady state concentration than lubricin following intra-articular lavage [50–52].

While intra-articular lavage resulted in loss of HA, either due to synovial fluid washout, increased degradation, or decreased production, it was surprising that lavage resulted in an increase in synovial fluid lubricin given prior in vitro data in bovine cartilage explants [13]. We hypothesized that intra-articular lavage would result in synovial fluid washout due to removal of synovial fluid from the joint, at least in the early post-lavage period. Unexpectedly, lubricin increased as early as 24 h post-lavage, peaking at 48 h post-lavage. The stimulus responsible for increased synovial fluid lubricin in these models is poorly understood and requires further exploration. One theory is that lubricin-coated white blood cells, such as peripheral mononuclear cells (PMNs), may contribute to the measured increases in synovial fluid lubricin by localizing to inflamed synovial fluid and joint tissues [53]. This theory would link the early increase in inflammatory cell-recruiting chemokines with the slightly delayed increase in lubricin in both intervention and control joints. However, the possibility of increased synovial membrane capillary permeability causing leakage of lubricin from the bloodstream, hemarthrosis as a result of arthrocentesis, and release from cartilage due to increased concentration of cartilage matrix degrading enzymes such as elastase [54, 55] cannot be ruled out based on the results of this study. Even so, this data suggests that lubricin may be a sensitive biomarker for joint inflammation.

IL-1β was chosen for the induction of synovitis as it induces a similar level of inflammation to equine synovial fluid as LPS and is a known contributor to the etiology of arthritis [32]. The absent response in IL-1β concentration and the brief TNF-α increase observed in IL-1β-induced synovitis joints differ from previous findings in the amphotericin B-induced synovitis model [37], which showed a sustained increase in concentration of these cytokines in synovial fluid for 9 weeks. Synovial fluid IL-1β protein concentrations have also been shown to increase in horses 8 h after LPS-induced synovitis [56] and in horses with naturally occurring OA [57]. The multiplex assay used in this study is capable of detecting *E. coli*-produced recombinant equine IL-1β. While there were no significant differences, there was a non-significant peak in IL-1β at 6 h post-induction of synovitis, which was likely residual recombinant IL-1β from the injection. Our data suggests that recombinant equine IL-1β is not auto-inductive despite resulting in significant elevations in TNF-α, CCL2, CCL3, CCL5, and CCL11. Increased IL-1β gene expression in synovium and articular cartilage has previously been reported in an equine IL-1β-induced synovitis model [58] and in naturally occurring osteoarthritis [59]; however, IL-1β gene expression does not necessarily correlate with bioactive IL-1β due to the requirement for post-translational modifications such as protease cleavage to result in the secreted, bioactive form of IL-1β [60]. Our sampling timeline for gene expression at day 30 may have missed the critical window to capture changes in IL-1β gene expression.

The synovial fluid chemokine results demonstrate the value of these inflammatory molecules as biomarkers following joint trauma. All 4 chemokine ligands rapidly increased by 6 h post induction of synovitis with no change in the contralateral MCJ, suggesting that repeated arthrocentesis alone did not induce these ligands. CCL2 and CCL11 showed mild increases in concentration in response to the lavage model that were not different from the contralateral TCJ. Whereas CCL2 and CCL3 returned to baseline levels within 24 h, CCL5 and CCL11 remained elevated from baseline for 72 h in the synovitis model. The data suggest that TNF-α, CCL2, CCL3, CCL5, and CCL11 are sensitive markers during early joint inflammation with variations in their temporal responses.

Repeated arthrocentesis is necessary for studies designed to measure changes in synovial fluid constituents over time and has been employed in nearly all studies evaluating IL-1β or LPS-induced synovitis in horse [32, 36, 37, 50, 52]. However, arthrocentesis is not an innocuous procedure and can lead to contamination of the joint with hair and debris, in addition to hemorrhage and trauma to the cartilage and synovium [61]. Increased joint circumference has been reported following 4 repeated arthrocenteses over a 24 h period in horses [62]. Several changes to synovial fluid composition have been documented following a single arthrocentesis in healthy equine and bovine joints, including elevations in matrix metalloproteinase 1, nitric oxide, PGE_2_, GAG [63, 64], and increased total leukocyte count [65]. In a previous study, HA content and synovial fluid viscosity as measured by the falling ball technique in equine middle carpal joints decreased following four repeated arthrocenteses over the course of 48 h [66]. Based on these findings, a sampling time of weekly or less frequently is ideal to prevent repeated arthrocentesis from affecting clinicopathologic outcome measures [63, 67]. However, synovitis models are transient, with most studies only measuring synovial fluid parameters over a 48 h time period, thereby necessitating more frequent sampling. Therefore, a larger number of study subjects sampled at less frequent intervals may be necessary to reduce the confounding effects of repeated sampling.

Synovial membrane biopsies were obtained at day 35 following induction of synovitis or joint lavage so that synovial fluid inflammatory and lubricating molecules could be studied without contaminating the joints with hemorrhage during the biopsy collection. While this study design was necessary to achieve the primary objectives of the study, 35 days is likely too late to capture the changes in synovial membrane morphology or gene expression induced by these transient models. Addition of more study subjects reserved for biopsy alone at earlier time points would be valuable to capture changes in gene expression and histologic changes. Interestingly, gene expression from synovial fluid cell pellets showed an increase in tumor necrosis factor-inducible gene-6 protein (*TSG6*) at 12- and 24-h post-induction of synovitis as compared to the lavage TCJ and increased expression at 24 h as compared to the control MCJ. TSG-6 is a protein with both anti- and pro-inflammatory effects, and its expression is induced by TNF-α [68]. Decreased expression of the hyaluronic acid synthases 1 and 3 in the synovitis MCJ compared to the lavage TCJ at 12 h post-induction suggesting a possible mechanism for the decreased HA concentration seen in this study, though *HAS2* is thought to be the primary contributor to synovial fluid HA expression [69].

An additional limitation to this study is that differences in baseline concentrations of hyaluronic acid in synovial fluid and viscosity exist between the MCJ and the TCJ which complicates the comparison between the synovitis and lavage models [47]; however, the TCJ is ideal for standing lavage and biopsy, and the use of both carpi and both tarsi enabled a randomized, crossover study design that required fewer horses. The multiplex assay used to test synovial fluid for inflammatory cytokines and chemokines is a new assay, and this is the first publication to describe the use of this assay on equine synovial fluid. While this limits the ability to directly compare to other studies, this assay enables assessment of 6 cytokines/chemokines simultaneously, maximizing valuable synovial fluid aliquots, and enables investigation into new potential biomarkers of joint disease and inflammation.

Additional studies are needed to elucidate the molecular mechanisms responsible for the increase in synovial fluid lubricin following repeated arthrocentesis. It is unclear whether synovial fluid lubricin is increased as a result of increased proteoglycan 4 expression in joint tissues, increased expression or release of lubricin from inflammatory WBCs or intra-articular hemorrhage, loss of lubricin from the articular cartilage surface or synovial lining, decreased metabolism/degradation of lubricin, or a combination of all of these mechanisms. One theory is that lubricin is released or upregulated as a protective mechanism for the joint in the face of inflammation and HA deficiency. In this way, lubricin may allow for continued joint lubrication, anti-adhesion and chondroprotection in situations when HA is decreased. Equine IL-1β synovitis and intra-articular lavage models are both non-terminal, transient models in which both inflammatory and lubricating molecules have been characterized and which may be valuable for investigations of anti-inflammatory and lubrication supplementation therapies.

## Conclusion

Contrary to previous in vitro reports, synovial fluid lubricin increases in response to IL-1β-induced synovitis and joint lavage in vivo. Both synovitis and lavage models induced changes in inflammatory and lubricating molecules, but the inflammatory changes were more pronounced in synovitis joints. Our data suggest that synovial fluid lubricin, in addition to a panel of synovial fluid biomarkers, including TNF-α, CCL2, CCL11 and sGAG, may have potential for elucidating the progression of early joint inflammation. Caution should be exercised when performing repeated arthrocentesis at frequent intervals, for either experimental research or clinical cases, due to the inflammatory response and loss of HA and synovial fluid viscosity associated with arthrocentesis.

## Methods

### Experimental design and sampling

Six horses, free from pathology affecting the middle carpal or tarsocrural joints, were enrolled in a complete block, randomized crossover study design. The experiment was designed to compare the clinical and synovial fluid biochemical and biophysical changes in response to IL-1β induced synovitis in the middle carpal joint (MCJ) and intra-articular lavage in the tarsocrural joint (TCJ). Breeds included Thoroughbred (2), Mustang/Arabian (2), and Warmblood (2), with ages ranging from 8 to 20 years (mean: 14.5 years, median: 15 years) and weights ranging from 511 to 636 kg (mean: 570 kg, median: 568.2 kg). An equal number of mares and geldings were included. Lameness and physical examinations were conducted prior to the study by two veterinarians to ensure that horses had an AAEP lameness grade of 2/5 or less, and carpal and tarsal radiographs were obtained to rule out any pre-existing joint pathology. Three of the horses had no lameness, two horses had a grade 1/5 lameness, and one horse had a grade 2/5 lameness. The horses all had minimal to no joint effusion in the MCJ or TCJ prior to commencement of the study. Treatment limbs were randomized using a Microsoft Excel random number generator with MCJ and TCJ groups being independent from each other. All horses received IL-1β induced synovitis in the MCJ first, followed by a washout period and the TCJ intra-articular lavage.

One hundred nanograms of recombinant equine IL-1β in 1 mL phosphate buffered saline (PBS) was administered into one randomly assigned middle carpal joint (MCJ), and the contralateral MCJ was injected with 1 mL PBS as a sham/vehicle injection. The recombinant equine IL-1β was all from the same lot number and was aliquoted on arrival to limit freeze thaw cycles. Synovial fluid (SF) and blood were collected at 0, 6, 12, 24, 48, 72, 168 (1 week), 336 (2 weeks), 504 (3 weeks), 672 (4 weeks), and 840 (5 weeks) hours post-induction of synovitis. At each time point, 3 mL of synovial fluid was collected, except the 840 h timepoint when as much fluid as possible was obtained. Following the last collection time point, a synovial membrane biopsy of the MCJ was performed under standing sedation using a small arthrotomy in the dorsolateral aspect of the MCJ and Ferris-Smith rongeurs. These incisions were closed in one layer of non-absorbable simple, interrupted sutures and were kept bandaged for 3 days following suture removal at day 14 post-procedure.

Following a 30-day wash-out period, which consisted of stall rest or small paddock turn-out, the same six horses entered the second stage of the study. This consisted of an intra-articular lavage performed under standing sedation via placement of four 14 g needles into each of the four quadrants of the tarsocrural joint (TCJ)—dorsomedial, dorsolateral, plantaromedial and plantarolateral. Lavage was performed using 2 L of Lactated Ringer’s Solution (LRS) into a randomly selected TCJ. The contralateral limb received no intervention. Synovial fluid and blood were collected over a 35-day period at the same time intervals as described for the synovitis intervention. Following the final collection timepoint at 840 h (5 weeks), a standing synovial membrane biopsy of the TCJ was obtained in four horses, while two horses were euthanized and samples were collected immediately post-mortem. Synovial fluid and blood were obtained ante-mortem.

All work was conducted under approval by the Institutional Animal Care and Use Committee (2018–0024). All joints were clipped and aseptically prepared prior to administration of recombinant IL-1β or intra-articular lavage, and synovial fluid sampling was performed using aseptic technique. One horse required general anesthesia for the intra-articular lavage due to intractable behavior.

### Evaluation of clinical response to treatment

A physical examination including heart rate, respiratory rate, rectal temperature, joint circumference, and range of motion, was performed at 0, 6, 12, 24, 48, 72, 168 (1 week), 336 (2 weeks), 504 (3 weeks), 672 (4 weeks), and 840 (5 weeks) hours post-induction of synovitis and lavage. A lameness examination at the walk and trot was performed at each time point, with a veterinarian assessing lameness at the walk and trot and an inertial sensor-based system (Equinosis Q) evaluating gait symmetry at the trot in hand on a firm, synthetic surface. For the synovitis model, bilateral carpal flexions were performed. No flexions were performed for the lavage model.

Joint circumference was measured with a tape measure at three locations for both carpi and tarsi, and the three measurements were averaged to obtain a single measure of effusion/edema. For the MCJ, circumference was measured at the accessory carpal bone (ACB), 2 cm distal to the ACB, and 4 cm distal to the ACB. For the TCJ, circumference was measured at the medial malleolus of the tibia, 1 cm distal to the malleolus, and 4 cm distal to the malleolus.

### Sample analysis

Synovial fluid was sampled/obtained prior to treatment (0 h), 6, 12, 24, 48, 72, 168 (1 week), 336 (2 weeks), 504 (3 weeks), 672 (4 weeks), and 840 (5 weeks) hours post-induction of synovitis or lavage. Arthrocentesis of 3 mL synovial fluid from either the MCJ or TCJ was performed aseptically following clinical assessment and lameness examination. Horses were sedated using either xylazine (0.2–0.4 mg/kg IV) or detomidine hydrochloride (0.004–0.016 mg/kg IV) combined with acepromazine maleate injection (0.1–0.2 mg/kg IV) and butorphanol tartrate (0.004–0.016 mg/kg IV). One horse required sedation with morphine sulfate (0.08–0.2 mg/kg IV) rather than butorphanol. Synovial fluid was placed in 15-mL conical polypropylene vials on ice and processed within 2 h of collection. A portion of the aspirate was used for analysis of total nucleated cell count and differential lymphocyte, monocyte, and neutrophil counts using an automated cell counter.

The remainder of the aspirate was centrifuged at 4000 x g for 15 min to remove cells and debris. The synovial fluid supernatants were aliquoted and stored at − 80 °C in 1.5 mL Eppendorf tubes. The cell pellet was re-suspended in 500 μL TRIzol Reagent and stored at − 80 °C. Jugular vein blood was collected into glass Vacutainer blood tubes with and without EDTA and centrifuged as above. Serum and plasma were aliquoted into 2 mL Eppendorf tubes and stored at − 80 °C. From the EDTA tubes, the buffy coat was aspirated from the red blood cell pellet and resuspended in 500 μL TRIzol Reagent and stored at − 80 °C. Once all SF samples were collected, they were assessed and scored subjectively for color/hemorrhage.

### BCA assay

A plate-based bicinchoninic acid assay (BCA, ThermoFisher Scientific, Waltham, MA) was performed to measure synovial fluid total protein concentrations colorimetrically [70]. Synovial fluid samples diluted in phosphate buffered saline (1:40 dilution) and a series of bovine serum albumin standards were loaded into a 96 well plate (Corning, Corning, NY) and incubated with BCA Working Reagent for 1 h at room temperature. Absorbance was measured on a plate reader at 562 nm (Tecan, Morrisville, NC).

### Synovial fluid lubricin (sandwich ELISA)

Synovial fluid lubricin concentration was measured at all time points by a sandwich ELISA using anti-lubricin monoclonal antibody 9G3 (MABT401; EMD Millipore, Darmstadt) and peanut agglutinin (PNA) (Sigma Aldrich, St. Louis, MO) as previously described [48, 71]. Briefly, after 12 h of coating at 4 °C with 10 mg/mL of PNA in 50 mM sodium bicarbonate buffer, pH 9.5, blocking was performed with (PBS) + 3% EIA-grade BSA (Sigma-Aldrich, St. Louis, MO) for 1 h. Equine purified lubricin standard and diluted equine synovial fluid samples (1:1000) were incubated for 1 h, the plate was washed with PBS + 0.1% Tween20, and monoclonal antibody 9G3 (mAbT401 Anti-Lubricin/Prg4 Clone 9G3) was loaded into the plate at 1:2500 dilution for 1 h. Following a wash cycle as above, goat anti-mouse IgG-horseradish peroxidase (EMD Millipore, Darmstadt, Germany) was added to each well at a 1:4000 dilution for 1 h. Washing three times in PBS + 0.1% Tween20, with a final rinse in PBS alone, was performed. TMB reagent was added (Pierce, Rockford, IL), the reaction was stopped with 1 N H2SO4, and absorbance was measured at 450 nm with 540 nm background subtraction. The intra-assay coefficient of variation for the lubricin assay was 11.6%, and the samples reading higher than the upper limit of detection of the assay due to oversaturation were assigned a value of 2000 μg/mL.

### Hyaluronic acid quantification – ELISA and gels

Synovial fluid HA concentration was measured at all time points using a commercially available HA ELISA (Hyaluronan DuoSet ELISA, Cat#: DY3614–05, R&D Systems, Minneapolis, MN) [72]. The distribution of HA molecular weights was determined by gel electrophoresis in a similar manner to that described previously [73]. Synovial fluid samples were diluted 1:15 with phosphate buffered saline and incubated overnight with 75 μg/ mL proteinase k (Proteinase K, recombinant, PCR grade, Roche Applied Science, Mannheim, Germany). Samples and standards, HiLadder (0.5–1.5 MDa) and Mega-HA Ladder (1.5–6.1 MDa; AMS Biotechnology Limited, Cambridge, MA) were loaded onto a 0.5% agarose gel and run at 57 V for 8 h. Gels were stained for 24 h in 0.005% Stains-All (Sigma-Aldrich, St. Louis, MO) in 50% ethanol and de-stained in 10% ethanol for 24 h with final destaining occurring on exposure to ambient light. Images of gels acquired using a Bio-Rad VersaDoc Imaging System (Hercules, CA) with relative band intensity calculated using Fiji Software (ImageJ). The intra-assay coefficient of variation for the HA ELISA was 9.1%.

### Histological processing

At day 35 post-induction of synovitis or post-lavage, a 2–3 mm sample of synovial membrane was obtained using a Ferris-Smith rongeur via standing arthrotomy from either the MCJ or the TCJ joint. Sections were fixed in 10% formaldehyde for a minimum of 3 days, dehydrated in alcohol, cleared in xylene, paraffin embedded and sectioned at 6 μm [74]. Slides were stained as one batch with haemotoxylin and eosin for basic cell identification, then evaluated by three blinded assessors.

### Chemokine multiplex assay

The equine chemokine multiplex assay has been validated and is performed at the Animal Health Diagnostic Center at Cornell University. The fluorescent bead-based assay simultaneously quantifies six equine cytokines/chemokines (IL-1β, TNF-α, CCL2, CCL3, CCL5, and CCL11) using pairs of monoclonal antibodies (mAbs) for detection of each equine chemokine. The procedures of coupling mAbs to the fluorescent beads (Luminex Corp., Austin, TX, USA) and performing the different steps of the assay were previously described in detail for other equine cytokines [75] and were identical for this assay. In brief, the following beads were coupled to mAbs: bead 33 with equine TNF-α mAb 292–1, bead 34 with equine CCL11 mAb 24, bead 35 with IL-1β mAb 84–2, bead 36 with CCL5 mAb 91–1, bead 37 with CCL2 mAb 104–2, and bead 42 with CCL3 mAb 77–2. Specificity to respective chemokine and recognition of the native proteins were confirmed for all mAbs [43] before they were used in the multiplex assay.

All six recombinant equine proteins were expressed in mammalian cells as IL-4 fusion proteins [43, 76]. For the assay runs, a mixture of the six recombinant chemokines was included in different concentrations (5-fold dilutions in PBS with 1% (w/v) BSA and 0.05% (w/v) sodium azide (blocking buffer)) to create standard curves for quantification of all six chemokines in equine samples. Joint samples were diluted 1:2 in blocking buffer. Millipore Multiscreen HTS plates (Millipore, Danvers, MA) were soaked with PBS with 0.1% (w/v) BSA, 0.02% (v/v) Tween 20 and 0.05% (w/v) sodium azide (PBS-T) using a ELx50 plate washer (Biotek Instruments Inc., Winooski, VT) for 2 min. The solution was aspirated from the plates and 50 μl of each diluted standard dilution or the samples were applied to the plates. Then, 50 μl of bead solution, containing 5 × 10^3^ beads per bead number, was added to each plate well and incubated with the standards or samples for 30 min on a shaker at room temperature. The plates were washed with PBS-T and 50 μl of the equine detection antibody mixture diluted in blocking buffer was added to each well and incubated for 30 min as above. The detection antibody mixtures included six biotinylated mAbs: TNF-α mAb 48–1, CCL11 mAb 25, IL-1β mAb 62–7, CCL5 mAb 46–1, CCL2 mAb 49, and CCL3 mAb 289–2 [43]. Afterwards plates were washed again and 50 μl of streptavidin-phycoerythrin (Invitrogen, Carlsbad, CA) was added to the plates for another 30 min incubation as above. Plates were washed for a last time, beads were resuspended in 100 μl of blocking buffer, and the plates were placed on the shaker for 15 min. The assay was analyzed in a Luminex 200 instrument (Luminex Corp., Austin, TX, USA). The data were reported as median fluorescent intensities. For standard curve fitting and subsequent calculation of the chemokine concentrations in samples the logistic 5p formula (y = a + b/(1 + (x/c)ˆd)ˆf) was used (Luminex 200 Integrated System). Chemokine concentrations were reported in pg/ml. For TNF-α and IL-1β the non-detectable values (0) were set to 1 for analysis.

### Dimethylmethylene blue (DMMB) assay

Synovial fluid samples were tested at all time points for sulfated glycosaminoglycan (sGAG) concentration using a 1,9-dimethylmethylene blue (DMMB) assay. DMMB dye was prepared by combining 16 mg DMMB dye (Sigma-Aldrich, St. Louis, MO) with 5 mL 95% ethanol and incubating at room temperature for 30 min. Two milliliters of pH 3.5 formate buffer was added to solution, and the total volume was adjusted to 1 L with water. The dye was stored at room temperature, protected from light. Synovial fluid samples were incubated at 37 °C with 30 U/mL Streptomyces hyaluronidase (Sigma-Aldrich, St. Louis, MO) for 1 h and were vortexed every 20 min during the digestion period. Digested samples were diluted to a final concentration of 1:30 in water. A solution of chondroitin 4-sulfate (Sigma-Aldrich, St. Louis, MO) was used as a standard in a range of concentrations from 2.5 μg/mL to 30 μg/mL. Milli-Q water was used as a blank. All samples, standards, and blanks were plated in duplicate at a volume of 50 μL on a transparent 96-well plate (Corning Inc., Corning, NY). After all samples were applied, the plate was agitated for 60 s on an orbital shaker (Bellco Glass Inc., Vineland, NJ). Two hundred microliters of DMMB dye was then added to each well of the plate using a multi-channel pipette, and absorbance was immediately read at 540 nm using a Spark 10 M plate reader (Tecan Austria GmbH, Grödig, Austria).

### PGE_2_ ELISA

Synovial fluid concentration of PGE_2_ was evaluated at all time points as previously described [30, 32]. In brief, 250 μL of synovial fluid (SF) was mixed with 250 μL of 80% ethanol and 5 μL of glacial acetic acid. After incubation, for 5 min at room temperature, and centrifugation (6000 rpm, 8 min), the supernatant was loaded onto Ethyl C2 mini-columns (Agilent Technologies, Santa Clara, CA) that had been equilibrated with 10% ethanol. The PGE_2_-containing mini-columns were washed with MQ-H_2_O and hexane sequentially. The PGE_2_ was eluted with two replicates of 375 μL of ethyl acetate. The combined 750 μL eluate was dried in a Speed Vacuum (Speed Vac Plus, SC110A, SAVANT) and the PGE_2_ powder was stored in at -70 °C. The PGE_2_ concentration in SF samples were measured with a highly sensitive and competitive PGE_2_ ELISA kit (Enzo Life Sciences, Inc., Farmingdale, NY). The PGE_2_ powder derived from 250 μL of synovial fluid was resuspended in 250 μL of PGE_2_ assay buffer. Then, 100 μL of the PGE_2_ solution was added in duplicate to the goat anti-mouse IgG microtiter plate. After being bound with the kit conjugate/antibody and wash, the plate was read at 405 nm with a background reading at 570 nm using SPARK 10 M microplate reader (TECAN, Zürich, Switzerland). The PGE_2_ levels in SF were evaluated using 4-parameter standard curve with the x-axis at Log scale. If the absorbance reading at 405 nm was above the standard range (maximum 2500 pg/mL), the PGE_2_ concentration was calculated as the maximum standard value (2500 pg/mL). The intra-assay coefficient of variation for the PGE_2_ ELISA was 7%.

### Microrheology

Synovial fluid viscosity was measured with particle tracking microrheology. 0.5 μm yellow-green fluorescent beads (FluoSpheres™ Carboxylate-Modified Microspheres, 0.5 μm, yellow-green fluorescent) were diluted in a 1:50 ratio with water. The diluted beads were then mixed with synovial fluid in a 1:50 ratio, totaling 20 μl. Samples were loaded into wells of silicone gaskets (Grace Bio-Labs Press-To-Seal silicone isolator, No PSA 24–2 mm diam. × 0.5 mm depth) which were press-sealed on 35 mm glass-bottom dishes (Cellvis D35–20-1.5-N). The sample was covered with a #1.5 glass coverslip to prevent evaporation and placed on an inverted fluorescence microscope (IX81, Olympus) equipped with a 60x NA 1.2 water-immersion objective and with 1x magnification. Fluorescence excitation by a 488 nm laser (Sapphire-LP, Coherent) was expanded 8.3x before focusing on the objective back aperture by a 300 mm tube lens (ThorLabs). Fluorescence emission was imaged with an EMCCD (897 Ultra, Andor) through a standard FITC filter set (Chroma) using the Micro-Manager software package (Open Imaging). Three 30-s videos were taken per sample, with several locations in each sample targeted to collect particle movement data. Each video contained approximately 15 particles in the frame. Analysis of motion was done using the Trackpy Python package. Images were acquired at 16 Hz.

### RNA extraction/gene expression

Gene expression was examined in synovial membrane (SM) samples of horses (*Equus caballus*) collected at day 35 post-induction of synovitis or intra-articular lavage and in synovial fluid cell pellets collected at 12- and 24 h post-induction. RNA was extracted from synovial membrane samples using the RNeasy Lipid Tissue Mini kit (QIAGEN, Gaithersburg, MD). The synovial fluid cell pellets were centrifuged as described above, the supernatant was aliquoted for freezing, and the SF cell pellet was suspended in 0.5 mL of TRIzol Reagent (ThermoFisher Sci., Waltham, MA). Crude RNA was extracted following the instruction manual of TRIzol Reagent. An RNA Clean and Concentrator kit (Zymo Research, Irvine, CA) was employed to further purify and concentrate the SF cell pellet RNA. Any remaining genomic DNA in the RNA extract was removed by DNase I digestion on-column for both SM and SF cell pellet RNA. RNA concentrations and quality were determined using a 16-well NanoQuant plate and a SPARK 10 M microplate reader (TECAN, Zürich, Switzerland). The expression levels of three genes (*PRG4*, *IL1β* and *HAS2* encoding for Hyaluronan synthase 2) in SM and four genes (*PRG4*, *TSG6* encoding for TNF-stimulated gene 6 protein, *HAS1* and *HAS3*) in SF cell pellets were analyzed.

Gene expression was detected by quantitative real-time PCR (qRT-PCR) using the Applied Biosystems Real-Time PCR ViiA 7 system (Applied Biosystems, Foster City, CA). All samples were analyzed in duplicate using the Power SYBR green RNA-to-C_T_ one-step kit (Applied Biosystem Inc., Carlsbad, CA). Primers (Supplemental Data 3) were selected from publications [48, 59] or designed by NCBI Primer 3 & Blast or with DNASTAR LASERGENE.

For qRT-PCR, 30 ng of SM total RNA or 15 ng of SF cell pellet RNA was used in 20 μL of reaction mix containing SYBR RT-PCR mix and RT enzyme mix. The qRT-PCR was run at 48 °C for 30 min and at 95 °C for 10 min, followed by 40 cycles of 95 °C/15 s and 60 °C/1 min. Successful qRT-PCR was verified via analysis of both dissociation curves and agarose gel electrophoresis. All values were normalized to the housekeeping gene 18S rRNA. Relative gene expression was analyzed using the 2^-∆CT^ method [77, 78], where ∆C_T_ = C_T_ (gene of interest)-C_T_ (18S rRNA) and calculated as (10^4^–10^7^) × 2^-∆CT^ [77].

### Statistical analysis

All analyses were conducted with Stata 16.1MP, StataCorp, College Station TX, with two-sided tests of hypotheses and a *p*-value < 0.05 as the criterion for statistical significance. Descriptive analyses include computation of means (with 95% confidence intervals [95%CI]), standard deviations, medians, interquartile ranges (IQR) of continuous variables and tabulation of categorical variables. Tests of normal distribution (Shapiro-Wilk test) were performed to determine extent of skewness. Frequency counts and percentages were used for categorical variables such as signalment and others.

Inference statistical analysis was based on a multilevel mixed-effects model with interaction between treatment group and categorical time as the fixed effects and age as a confounder. Random effects were set on the level of joint nested within leg which in term was nested within specific animal. All random effects were considered random intercepts. Robust estimation of the variance was used to permit for departures from normality of the outcome. *Post-hoc* pairwise comparisons were conducted to estimate the marginal (model adjusted) effects. Least significant difference (LSD) was used to adjust for multiple comparisons. The figures presented in this paper depict the marginal means used in the statistical model and not the raw data which is presented in Supplemental Data 2.

## Supplementary Information

**Additional file 1: Supplemental Data 1.** Heart rate (HR), temperature (T), respiratory rate (RR), and joint circumference (JC) following IL-1β-induced synovitis or intra-articular lavage. Graphs depicting changes in heart rate, temperature, respiratory rate and joint circumference following induction of synovitis and intra-articular lavage.

**Additional file 2: Supplemental Data 2.** Mean and standard error of the mean for the non-adjusted parameters measured in synovial fluid following synovitis induction and intra-articular lavage of the MCJ and TCJ. Data table of the means and standard error of the means for the unadjusted data described in the paper.

**Additional file 3: Supplemental Data 3.** Equine gene names, accession numbers, primer sequences and amplicon sizes for qRT-PCR. Table of primers used for gene expression analysis used in this paper.

## Data Availability

The datasets used and/or analyzed during the current study are available from the corresponding author on reasonable request.

## Abbreviations

OA: Osteoarthritis
IL-1β: Interleukin 1 beta
CCL: Chemokine receptor ligand
HA: Hyaluronic acid
TNFα: Tumor necrosis factor alpha
PGE_2_: Prostaglandin E_2_
SF: Synovial fluid
SM: Synovial membrane
LPS: Lipopolysaccharide
MMPs: Matrix metalloproteases
ADAMTS4: A disintegrin and metalloproteinase with thrombospondin motif 4
TP: Total protein
sGAG: Sulfated glycosaminoglycans
ELISA: Enzyme linked immunosorbent assay
RT-PCR: Reverse transcriptase polymerase chain reaction
PMN: Polymorphonuclear cells
WBC: White blood cells
TCJ: Tarsocrural joint
MCJ: Middle carpal joint
AAEP: American Association of Equine Practitioners
PBS: Phosphate buffered saline
ACB: Accessory carpal bone
PNA: Peanut agglutinin
HR: Heart rate
T: Temperature
HMW HA: High molecular weight hyaluronic acid
TSG6: Tumor necrosis factor-stimulated gene-6 protein
HAS1–3: Hyaluronan synthase 1–3
