# Supplementary material for: Investigation of synovial fluid lubricants and inflammatory cytokines in the horse: a comparison of recombinant equine interleukin 1 beta-induced synovitis and joint lavage models
Source: BMC Vet Res. 2021 May 12;17:189. doi: 10.1186/s12917-021-02873-2 (PMC8117281; doi:10.1186/s12917-021-02873-2)
Supplement: Supplementary file 1 — Additional file 1: Supplemental Data 1. Heart rate (HR), temperature (T), respiratory rate (RR), and joint circumference (JC) following IL-1β-induced synovitis or intra-articular lavage. Graphs depicting changes in heart rate, temperature, respiratory rate and joint circumference following induction of synovitis and intra-articular lavage. [file 12917_2021_2873_MOESM1_ESM.pdf]

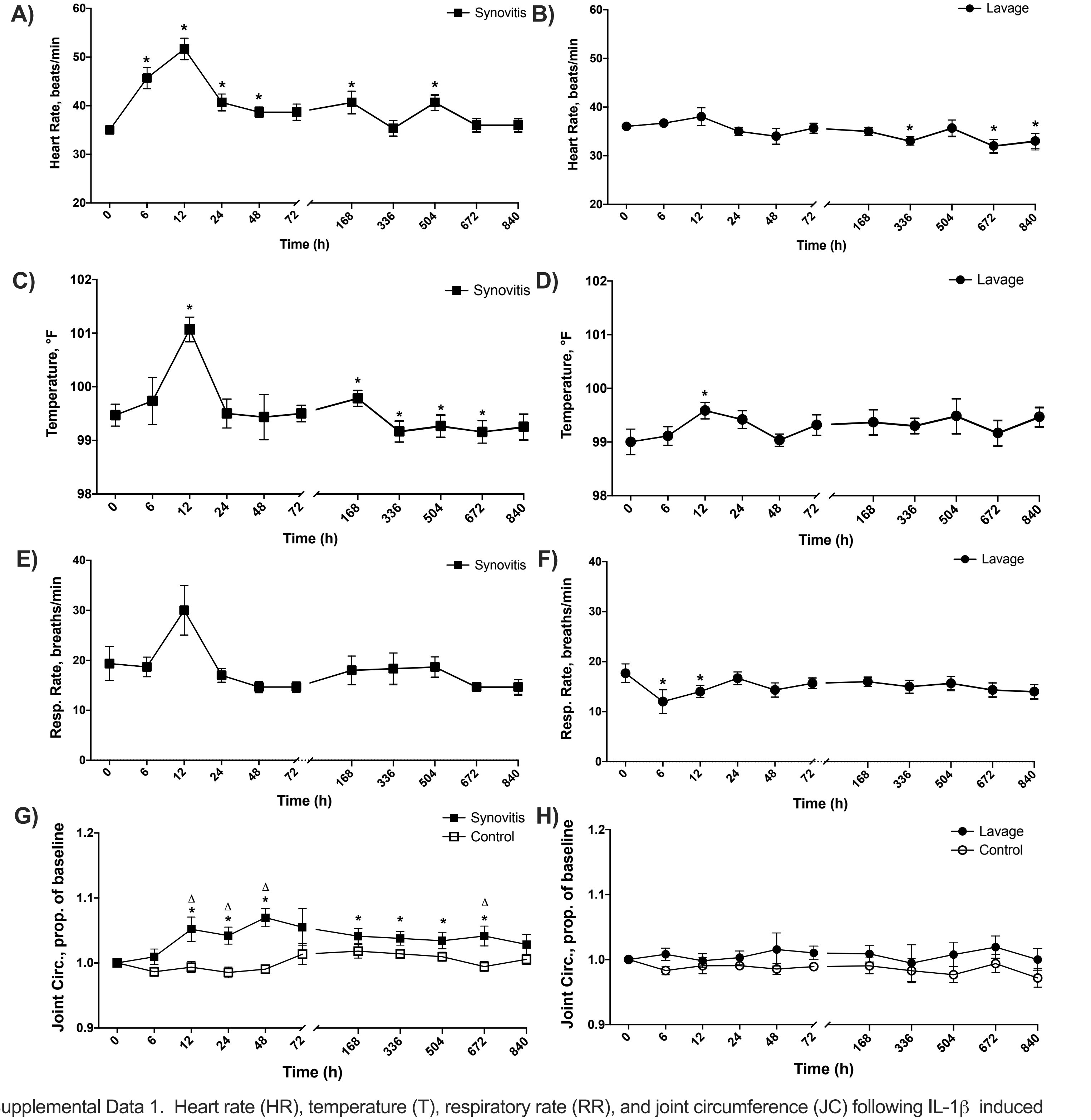

Supplemental Data 1. Heart rate (HR), temperature (T), respiratory rate (RR), and joint circumference (JC) following IL-1β induced synovitis or intra-articular lavage. HR is increased from baseline for 1 week following synovitis induction and again at 3 weeks (A). HR is unchanged following lavage apart from a decrease seen at 2, 4, and 5 weeks (B). T is increased from baseline at 12 hours post-synovitis. An increase is seen at 1 week and decreases at 2, 3, and 4 weeks were seen (C). T shows a weak increase at 12 hours post-lavage (D). RR is unchanged following synovitis (E). RR is decreased following lavage at 6 and 12 hours (F). JC, shown as a proportion of baseline, is increased from baseline for 4 weeks following synovitis and was greater in the synovitis MCJ than the contralateral MCJ for 48 hours (G). JC was unchanged following lavage (H).

* - Significant difference from baseline

Δ - Significant difference from contralateral limb
