# Supplementary material for: Investigation of synovial fluid lubricants and inflammatory cytokines in the horse: a comparison of recombinant equine interleukin 1 beta-induced synovitis and joint lavage models
Source: BMC Vet Res. 2021 May 12;17:189. doi: 10.1186/s12917-021-02873-2 (PMC8117281; doi:10.1186/s12917-021-02873-2)
Supplement: Supplementary file 2 — Additional file 2: Supplemental Data 2. Mean and standard error of the mean for the non-adjusted parameters measured in synovial fluid following synovitis induction and intra-articular lavage of the MCJ and TCJ. Data table of the means and standard error of the means for the unadjusted data described in the paper. [file 12917_2021_2873_MOESM2_ESM.pdf]

Supplemental Data 2: Mean and standard error of the mean for the non-adjusted parameters measured in synovial fluid following synovitis induction and intra-articular lavage of the MCJ and TCJ.

			**Timepoint (hr)**	**0**	**6**	**12**	**24**	**48**	**72**	**168**	**336**	**504**	**672**	**840**
**TP (g/dL)**	Synovitis	Affected	Mean	2.0	5.6	5.7	6.4	5.1	4.5	2.8	2.1	1.7	1.5	1.5
			SEM	0.3	0.5	0.5	0.3	0.5	0.2	0.7	0.4	0.4	0.3	0.3
		Control	Mean	1.9	2.0	2.4	3.4	3.2	2.6	1.7	1.8	1.7	1.4	1.5
			SEM	0.2	0.3	0.4	0.5	0.5	0.5	0.3	0.4	0.3	0.2	0.3
	Lavage	Affected	Mean	1.7	2.4	2.7	2.9	2.4	2.3	1.5	1.4	1.1	1.3	1.5
			SEM	0.1	0.5	0.5	0.4	0.2	0.1	0.2	0.2	0.1	0.1	0.5
		Control	Mean	2.1	3.5	3.1	3.4	2.8	2.9	1.6	1.5	1.4	1.3	1.3
			SEM	0.4	0.5	0.3	0.4	0.3	0.5	0.3	0.2	0.2	0.1	0.1
**WBC (cells/µL)**	Synovitis	Affected	Mean	0.7	97.4	72.1	56.0	27.1	13.7	1.4	0.7	0.4	0.5	0.7
			SEM	0.3	24.2	12.7	7.4	5.9	3.2	0.2	0.1	0.1	0.1	0.3
		Control	Mean	0.3	1.2	6.3	5.1	5.2	2.5	0.5	0.6	0.4	0.4	0.5
			SEM	0.1	0.5	2.7	1.7	2.5	0.9	0.0	0.2	0.1	0.1	0.2
	Lavage	Affected	Mean	0.9	9.8	11.8	6.9	4.1	3.8	0.9	0.4	0.4	0.4	1.1
			SEM	0.5	2.7	3.0	0.5	0.5	0.9	0.2	0.1	0.1	0.1	0.6
		Control	Mean	0.5	5.6	5.3	5.2	4.1	3.2	0.8	0.4	0.3	0.4	0.4
			SEM	0.1	3.3	2.8	2.2	0.9	0.9	0.1	0.0	0.0	0.1	0.1
**PGE_2 (pg/mL)**	Synovitis	Affected	Mean	72.9	2179.9	2134.3	670.2	538.4	497.6	245.1	124.5	68.0	78.9	53.1
			SEM	18.2	292.3	333.8	259.3	358.7	223.5	97.2	39.0	18.9	37.2	20.6
		Control	Mean	44.5	66.3	89.8	39.6	49.2	81.5	46.4	65.7	56.1	32.8	48.4
			SEM	9.4	5.5	20.5	5.1	9.6	19.8	7.6	33.4	24.8	4.5	17.0
	Lavage	Affected	Mean	61.3	339.3	359.6	93.1	57.3	68.1	66.5	115.5	61.7	40.6	30.9
			SEM	27.6	116.8	182.3	24.9	8.6	21.8	12.4	53.8	19.2	7.3	4.6
		Control	Mean	91.7	134.2	106.5	64.7	51.3	53.2	137.3	69.2	36.3	45.0	39.0
			SEM	36.4	44.3	29.1	20.8	8.4	10.6	70.0	19.7	5.5	7.2	9.7
**HA (mg/mL)**	Synovitis	Affected	Mean	0.6	0.3	0.4	0.4	0.5	0.3	0.3	0.4	0.3	0.3	0.3
			SEM	0.1	0.1	0.1	0.1	0.0	0.0	0.1	0.1	0.1	0.0	0.1
		Control	Mean	0.5	0.3	0.2	0.3	0.4	0.4	0.2	0.3	0.2	0.3	0.4
			SEM	0.1	0.0	0.0	0.1	0.1	0.1	0.0	0.1	0.0	0.0	0.1
	Lavage	Affected	Mean	0.4	0.2	0.3	0.4	0.5	0.4	0.3	0.3	0.3	0.4	0.3
			SEM	0.1	0.0	0.0	0.0	0.0	0.0	0.0	0.0	0.0	0.0	0.0
		Control	Mean	0.5	0.4	0.4	0.5	0.5	0.5	0.3	0.3	0.3	0.4	0.3
			SEM	0.0	0.0	0.0	0.1	0.1	0.0	0.0	0.0	0.0	0.1	0.0

			Timepoint (hr)	0	6	12	24	48	72	168	336	504	672	840
**Viscosity (cP)***	Synovitis	Affected	Mean	84.8			25.3		24.1	14.6			29.0	
			SEM	13.5			2.6		4.5	1.9			4.7	
		Control	Mean	96.5			70.1		67.3	33.9			47.1	
			SEM	16.1			7.1		8.8	7.9			7.1	
	Lavage	Affected	Mean	17.3			28.2		17.4	9.4			12.0	
			SEM	2.0			2.7		2.5	0.5			1.1	
		Control	Mean	20.3			40.4		22.9	11.7			13.4	
			SEM	2.9			6.4		2.2	1.2			2.2	
**Lubricin (µg/mL)**	Synovitis	Affected	Mean	77.2	113.6	183.2	505.4	1336.9	1259.2	673.1	196.0	118.7	155.5	99.9
			SEM	23.7	29.4	42.8	93.4	208.5	240.9	104.9	42.3	49.5	58.2	27.6
		Control	Mean	74.8	72.7	107.3	706.3	1227.1	925.9	266.1	140.9	234.3	128.3	113.2
			SEM	20.1	15.3	35.2	167.5	287.9	308.3	102.5	32.3	143.7	52.1	25.0
	Lavage	Affected	Mean	86.7	72.1	112.7	1402.0	1747.3	1259.7	670.1	219.7	119.9	62.3	77.6
			SEM	36.9	28.2	19.0	136.9	80.0	197.9	219.1	75.8	36.7	22.0	22.1
		Control	Mean	94.2	106.4	152.0	888.2	1371.1	1369.0	763.5	239.9	99.8	177.8	66.2
			SEM	46.7	48.7	56.4	230.4	235.3	269.7	212.9	111.1	45.3	81.7	26.7
**sGAG (µg/mL)**	Synovitis	Affected	Mean	388.5	500.6	559.7	701.8	726.4	629.3	421.7	340.9	311.7	323.3	317.7
			SEM	35.6	33.8	13.7	17.0	29.2	42.9	36.2	42.2	27.4	36.3	36.8
		Control	Mean	457.2	397.5	453.1	601.3	445.0	360.2	357.9	331.3	337.5	369.3	417.3
			SEM	45.4	31.6	50.7	70.2	52.1	63.7	69.1	45.0	51.6	56.9	73.5
	Lavage	Affected	Mean	278.4	322.2	317.9	401.3	388.7	383.8	270.7	243.9	232.3	214.0	252.4
			SEM	24.5	33.4	19.4	30.7	41.6	39.6	24.3	24.3	29.7	29.7	45.1
		Control	Mean	309.9	378.1	340.3	481.6	433.9	397.0	295.4	259.5	250.2	294.5	302.4
			SEM	36.8	47.3	31.3	40.2	24.4	10.9	21.9	35.1	33.9	35.9	26.2
**CCL2 (pg/mL)**	Synovitis	Affected	Mean	401.8	94021.8	32555.3	44933.3	15540.2	6935.8	1754.5	721.7	637.5	342.2	377.7
			SEM	160.1	43243.8	14203.4	17547.6	6536.1	3588.0	1065.1	136.5	112.6	57.2	29.7
		Control	Mean	328.3	1492.5	10004.5	3596.3	4147.7	1234.5	380.5	541.5	371.2	358.5	2577.0
			SEM	98.7	686.6	3613.3	1203.0	1728.5	416.4	84.4	190.8	125.0	86.8	2149.0
	Lavage	Affected	Mean	132.2	38147.0	21701.0	1463.0	553.2	388.8	412.8	324.2	298.5	302.2	386.7
			SEM	30.0	12903.9	12313.1	163.3	65.4	50.1	89.2	31.9	57.5	46.2	139.1
		Control	Mean	329.8	20503.8	18547.5	1463.8	670.3	665.0	652.5	409.3	387.8	764.8	287.5
			SEM	122.1	16785.7	7552.8	365.2	168.6	164.5	224.4	59.6	71.4	302.6	64.5

**Timepoint (hr)**				**0**	**6**	**12**	**24**	**48**	**72**	**168**	**336**	**504**	**672**	**840**
**CCL3 (pg/mL)**	Synovitis	Affected	Mean	73.2	2217.5	553.7	266.3	368.5	637.3	363.5	212.8	145.0	39.7	88.7
			SEM	66.8	1815.5	505.4	243.1	212.8	368.1	253.9	194.3	132.4	36.2	80.9
		Control	Mean	184.7	120.5	174.7	149.0	214.0	337.2	27.0	102.0	148.0	80.2	215.5
			SEM	168.6	110.0	159.4	88.1	195.4	207.9	24.6	93.1	135.1	73.2	196.7
	Lavage	Affected	Mean	6.0	164.8	186.2	0.0	0.0	0.0	77.2	92.8	38.7	59.8	0.0
			SEM	5.5	131.6	134.1	0.0	0.0	0.0	70.4	84.7	35.3	54.6	0.0
		Control	Mean	1157.7	259.7	58.7	94.3	0.0	123.3	71.8	89.8	68.7	109.8	72.5
			SEM	920.6	209.2	53.6	86.1	0.0	112.6	65.6	82.0	62.7	100.3	66.2
**CCL5 (pg/mL)**	Synovitis	Affected	Mean	166.5	634.0	421.0	370.5	378.5	539.7	361.5	203.7	238.8	120.8	190.3
			SEM	152.0	474.3	359.0	262.1	207.7	315.1	211.2	183.2	205.5	110.3	173.7
		Control	Mean	222.7	160.2	205.0	142.3	170.8	194.2	62.2	156.8	216.3	189.0	242.8
			SEM	203.3	146.2	175.9	129.9	137.7	177.2	56.8	143.2	197.5	159.1	221.7
	Lavage	Affected	Mean	98.3	199.3	197.0	117.0	88.0	117.8	136.2	144.7	131.5	142.0	109.2
			SEM	89.8	154.0	158.6	74.1	64.0	107.6	124.3	132.1	116.6	129.6	97.5
		Control	Mean	195.3	108.7	157.0	156.7	84.2	89.5	176.5	148.8	180.5	219.5	163.7
			SEM	115.6	64.7	134.2	122.9	76.8	76.5	157.7	135.9	151.5	180.7	145.2
**CCL11 (pg/mL)**	Synovitis	Affected	Mean	3178.8	16159.7	14109.3	13242.0	13004.7	9195.0	7352.7	4027.8	3938.3	2655.3	3276.7
			SEM	1526.8	6748.9	6648.1	6136.9	5170.4	3883.3	3381.0	1827.4	1966.7	1065.1	1313.8
		Control	Mean	3696.8	3197.5	4370.5	4113.8	6370.0	6228.2	2563.8	3843.8	3612.7	3407.2	7736.2
			SEM	1998.8	1368.8	1626.8	1055.1	2401.8	3270.7	968.2	1779.7	2129.2	1486.1	5431.9
	Lavage	Affected	Mean	1841.2	9817.5	6916.7	4766.7	2386.5	2527.3	2403.8	2615.2	2567.2	2235.8	2937.3
			SEM	392.6	3209.8	1160.3	1532.2	480.6	140.6	374.6	544.5	466.5	726.6	637.2
		Control	Mean	5605.8	7032.5	6723.7	4737.5	2479.2	4014.0	3202.0	3117.7	2694.8	3382.0	4081.7
			SEM	2731.0	1845.2	1410.7	836.3	332.6	604.7	855.6	770.1	675.1	1049.3	1308.6
**TNFα (pg/mL)**	Synovitis	Affected	Mean	5.0	4283.2	849.3	290.3	372.2	480.8	209.2	0.0	0.0	0.0	0.0
			SEM	4.6	2742.6	405.8	139.9	191.8	309.2	190.9	0.0	0.0	0.0	0.0
		Control	Mean	40.0	0.0	2.7	100.5	106.8	51.2	0.0	0.0	0.0	0.0	57.0
			SEM	36.5	0.0	1.8	91.7	66.6	46.7	0.0	0.0	0.0	0.0	52.0
	Lavage	Affected	Mean	0.0	0.0	0.0	46.7	7.8	0.0	0.0	0.0	0.0	0.0	0.0
			SEM	0.0	0.0	0.0	42.6	7.2	0.0	0.0	0.0	0.0	0.0	0.0
		Control	Mean	0.0	0.0	0.0	0.0	0.0	0.0	0.0	0.0	0.0	0.0	0.0
			SEM	0.0	0.0	0.0	0.0	0.0	0.0	0.0	0.0	0.0	0.0	0.0
**IL-1β (pg/mL)**	Synovitis	Affected	Mean	730.5	2545.0	652.3	0.0	45.5	1279.0	896.0	645.5	903.8	413.8	869.7
			SEM	666.9	1948.5	595.5	0.0	41.5	832.1	551.3	589.1	825.1	377.8	793.9
		Control	Mean	1213.8	897.0	775.7	232.0	343.3	197.8	59.8	510.2	914.2	787.8	66.8
			SEM	1108.1	607.5	708.1	211.8	313.4	180.6	54.6	465.7	834.5	719.2	61.0
	Lavage	Affected	Mean	301.3	1009.8	948.3	105.3	130.8	269.0	699.5	796.2	520.2	565.8	446.0

			SEM	275.1	921.8	865.7	96.2	119.4	245.6	638.6	726.8	474.8	516.5	407.1
		Control	Mean	471.3	143.3	754.7	465.7	102.0	186.7	698.2	694.0	828.2	1012.5	699.5
			SEM	430.3	130.8	688.9	425.1	93.1	170.4	637.3	633.5	756.0	924.3	638.6

* Viscosity measurements were limited to 5 timepoints
