# Supplementary material for: Investigation of synovial fluid lubricants and inflammatory cytokines in the horse: a comparison of recombinant equine interleukin 1 beta-induced synovitis and joint lavage models
Source: BMC Vet Res. 2021 May 12;17:189. doi: 10.1186/s12917-021-02873-2 (PMC8117281; doi:10.1186/s12917-021-02873-2)
Supplement: Supplementary file 3 — Additional file 3: Supplemental Data 3. Equine gene names, accession numbers, primer sequences and amplicon sizes for qRT-PCR. Table of primers used for gene expression analysis used in this paper. [file 12917_2021_2873_MOESM3_ESM.pdf]

Supplemental Data 3. Equine gene names, accession numbers, primer sequences and amplicon sizes for qRT-PCR

Genes	Accession number	Primer sequences	Amplicon size (bp)
PRG4	XM_023640721.1	For: AAACAGGAACCCATCAGAAAGTG<br>Rev: TGCGGTGCTTCCCCATAC	74
IL1β	XM_001495926.5	For: CGTCTCCCAGAGCCAATCC<br>Rev: CACCAGGCTGACTTTGAGTGAGT	83
HAS1	XM_023650323.1	For: GCGATACTGGGTGGCCTTCAATGT<br>Rev: CTGTATAGGCCTAGGGGACCACTG	90
HAS2	NM_001081801.2	For: GGCCGGTCGTCTCAAATTCA<br>Rev: TCACAATGCATCTTGTTCAGCTC	132
HAS3	XM_023637194.1	For: CGTGGGCGCATCTGGAACATT<br>Rev: CTCTGCATTGCCCCGAAGGAAG	99
***18S* rRNA**	NR_046271.1	For: GGCGTCCCCCAACTTCTT<br>Rev: AGGGCATCACAGACCTGTTATTG	77
TSG6	NM_001081906.1	For: ATCCTGAGCAGCCCCTAACA<br>Rev: TTGAATCCCCATCCGTGAGC	108
